# Effects of different sequences of pulmonary artery and vein ligations during pulmonary lobectomy on blood micrometastasis of non-small cell lung cancer

**DOI:** 10.3892/ol.2012.1022

**Published:** 2012-11-09

**Authors:** PING-PING SONG, WEIDI ZHANG, BAIJIANG ZHANG, QI LIU, JIAJUN DU

**Affiliations:** 1 Institute of Oncology, Provincial Hospital Affiliated to Shandong University, Shandong University, Jinan 250021;; 2 Department of Thoracic Surgery, Shandong Tumor Hospital, Jinan 250117, P.R. China

**Keywords:** lung cancer, blood, micrometastasis

## Abstract

The aim of this study was to investigate the effects of different sequences of pulmonary artery and vein ligations during lobectomy on blood micrometastasis of non-small cell lung cancer (NSCLC). Cytokeratin 19 (CK19)/adhesion molecule CD44v6 mRNA were used as markers. A total of 30 NSCLC patients undergoing pulmonary lobectomy were randomly divided into pulmonary artery (PA)-first and pulmonary vein (PV)-first groups according to the order of artery or vein ligation (15 cases in each). Fluorescent quantitative-RT-PCR (FQ-RT-PCR) was used to detect the mRNA expression of CK19 and CD44v6 in pulmonary venous blood at the early and late periods during surgery, and ΔCt values were calculated. Meanwhile, the peripheral blood samples from 10 healthy volunteers were selected as the control. ΔCt values of CD44v6 and CK19 of NSCLC groups at the early period during surgery were 7.83±1.70 and 10.76±2.74, while those of the control group were 9.17±1.04 and 12.76±2.36. The expression of CD44v6 and CK19 genes in venous blood of NSCLC groups was significantly higher than that of the control group (P<0.05). In addition, the ΔCt values of CD44v6 and CK19 in the early and late periods during surgery in the PA-first group were 7.92±1.97 vs. 5.67±2.11 (P= 0.008) and 11.21±3.14 vs. 8.60±4.02 (P= 0.05), respectively. The expression of CD44v6 and CK19 in the late period were both significantly higher than those in the early period, while neither the ΔCt value of CD44v6 nor that of CK19 in the early vs. late periods in the PV-first group exhibited statistically significant differences (7.95±1.91 vs. 7.74±2.10 and 10.60±3.15 vs. 10.30±2.98) (P<0.05). Surgical manipulation itself may stimulate the occurrence of blood micrometastasis and the ligation of the PV first during surgery may help prevent blood micrometastasis.

## Introduction

Circulating tumor cells (CTCs) are present in the blood of patients with malignant tumors and are strongly correlated with the prognosis of patients. CTCs are formed when certain tumor cells escape from the solid tumor cell nests and gain access to the blood circulation. Thus, it is likely that any touching or crushing of the tumor during surgery may initiate this process. The issue of whether surgical manipulation of pulmonary lobectomy promotes blood micrometastasis, and whether different sequences of pulmonary artery (PA) and vein (PV) ligations have different effects on blood micrometastasis of pulmonary carcinoma remain controversial. Kurusu *et al* observed the mRNA expression of carcino-embryonic antigen (CEA) in the blood at early, middle and late periods during surgery and concluded that surgery could promote blood micrometastasis (1). Conversely, Refaely concluded that different sequences of PA and PV ligations bore no correlation with the prognosis of the patients (2). However, in the previous studies, the comparisons of related gene expression were made between those in the pulmonary venous blood during surgery and those in the peripheral blood before and after surgery, and thus certain errors may have been caused due to different blood samples. Therefore, to clarify the investigation of whether different sequences of PA and PV ligations have different effects on blood micrometastasis, errors caused by different blood samples should be avoided by making a comparison between gene expression in pulmonary venous blood at early and late periods during surgery (3). Thus, to perform a rigorous comparison, blood samples at early and late periods during surgery should both be drawn from the PV.

In this study, we aimed to assess whether the process of surgical manipulation itself affects blood micrometastasis. We randomly divided 30 patients suffering from non-small cell lung cancer (NSCLC) into PA-first and PV-first groups. Fluorescent quantitative-RT-PCR (FQ-RT-PCR) was used to detect the mRNA expression of CK19 and CD44v6 in the pulmonary venous blood at early and late periods during each operation.

## Materials and methods

### Patients

From January 2009 to January 2010, a total of 30 NSCLC patients underwent lobectomy for NSCLC in the thoracic surgery department of Shandong Tumor Hospital, China. The post-operative pathologic tumor-node-metastases (TNM) stage was determined according to the grading criteria of UICC. The diagnosis of lung cancer was histologically confirmed on each resected specimen where the tumor size was measured. Eligible patients had no history of previous anticancer therapy (including radiotherapy and chemotherapy) or other malignancy. The 10 cases in the control group were healthy volunteers with no history of malignancy. The study was approved by the ethics committee of Shandong Cancer Hospital and Institute, Jinan, China. Written informed patient consent was obtained from the patient’s family with warrant of attorney.

### Blood samples

All NSCLC patients were randomly divided into two groups: PV-first (15 cases) and PA-first (15 cases). For the PV-first group, the PV was separated following anesthesia and opening of the chest, 10 ml blood was drawn from the proximal part and then the vein was immediately ligated (but not cut off) at the distal part. Then, routine operative procedures were performed. After 30 min, another 10 ml blood was drawn from the proximal part of the PV (as distant from the ligated spot as possible) and then the vein was cut off. For the PA-first group, the PV was separated following anesthesia and opening of the chest, 10 ml blood was drawn from the proximal part and soon afterwards the ligation of the PA was carried out. After 20–45 min, 10 ml blood was drawn from the proximal part of the vein. All patients underwent routine mediastinal lymphadenectomy following pulmonary lobectomy and recovered well after the operation. Peripheral vein blood samples were drawn from the controls. All blood samples were added into EDTA anticoagulation tubes and blood monocytes were obtained by Ficoll density gradient centrifugation, rinsing with PBS and centrifuging twice. Then, they were dissolved by adding 1 ml TRIzol reagent for the extraction of total RNAs.

### Extraction of total RNAs

Total RNAs were extracted by adding 0.25 ml TRIzol™ reagent to Ficoll-isolated cells, mixing thoroughly and maintaing at room temperature for 5 min. Then, the homogenate was transferred into a 1.5 ml EP tube to which 0.05 ml choloroform was added, shaken vigorously for 15 sec and then maintained at room temperature for 2–3 min. This was centrifuged at 10,000 × g for 15 min at 2–8°C. The upper aqueous phase in the EP tube was transferred into a fresh tube, to which 0.125 ml isopropanol was added, and then maintained at room temperature for 10 min. This was centrifuged at 10,000 × g for 10 min at 2–8°C. Supernatants were discarded. RNA precipitate was washed twice with 0.5 ml 75% ethanol, centrifuged at 7,500 × g for 5 min at 2–8°C, dehumidified in air and then dissolved by adding RNase-free H_2_O. The absorbance of 260 nm (A260) was measured by nucleoprotein quantitative analyzer and the ratio of A260/A280 was used to measure RNA purity (the ratios were 1.8/2.0 in this study). All the samples were preserved at −80°C.

### Real-time PCR

RT reaction was carried out using PrimeScript™ RT kit (Perfect Real Time, Applied Biosystems, Carlsbad, CA, USA). The reaction system (10 *μ*l) contained 2 *μ*l 5X PrimeScript Buffer, 0.5 *μ*l PrimeScript RT Enzyme Mix I, 0.5 *μ*l Oligo dT Primer (50 *μ*M), 0.5 *μ*l random 6 primer (100 *μ*M) and 1 *μ*g RNA. The reaction conditions were 37°C for 15 min, followed by 85°C for 5 sec. Subsequently, cDNA samples were preserved in a −80°C refrigerator.

SYBR^®^ Premix Ex Taq™ (Perfect Real Time) kit was used for real-time PCR. A quantitative PCR system (25 *μ*l) contained 12.5 *μ*l 2X SYBR Premix Ex Taq, 0.5 *μ*l forward primer (10 *μ*M), 0.5 *μ*l reverse primer (10 *μ*M) and 100 ng cDNA. The primer sequences of CD44v6 were 5′-AGCAACCCTACTGATGATGACG-3 (forward) and 5′-GGAGTCTTCTCTGGGTGTTTGG-3 (reverse), and those of CK19 were 5′-CTTCCGAACCAAGTTTGAGACG-3 (forward) and 5′-CCTCAGCGTACTGATTTCCTCCT-3 (reverse). An ABI PRISM 7700 system was used for PCR. The amplification conditions were a cycle of pre-denaturation at 95°C for 30 sec, 40 cycles of 95°C for 5 sec and 60°C for 30 sec. β-actin was used as an internal standard. All the samples were repeated three times and the values of ΔCt were calculated. The Ct value was calculated by determining the point at which the fluorescence exceeded an arbitrary threshold limit. The value of ΔCt was obtained from the formula: Ct = Ct (target gene) − Ct (β-actin). A higher ΔCt value indicated a lower target gene content and gene expression was lower with more amplification. Conversely, a lower ΔCt value indicated a greater target gene content and gene expression was higher with less amplification.

### Statistical analysis

The Ct data of the genes were analyzed by SDS software (ABI). All the data were analyzed by SPSS 11.5 software. Student’s t test was used. Differences were considered significant when P<0.05.

## Results

### mRNA expression of CD44v6 and CK19

**Figure 1.**
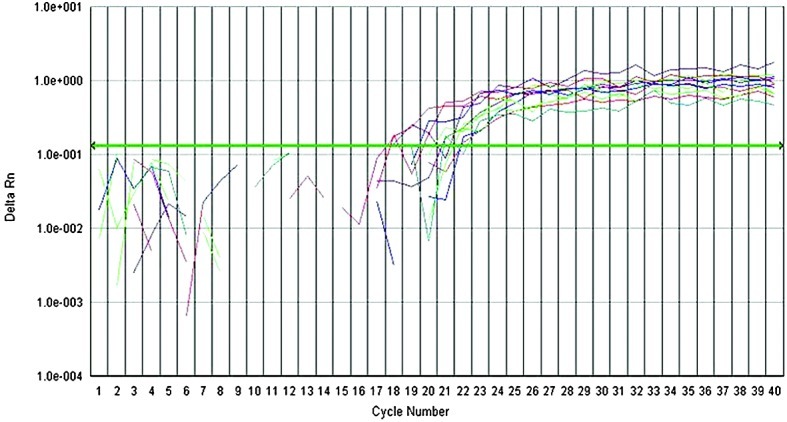
Fluorescent quantitative RT-PCR amplification curves of β-actin. The graph of the increment of fluorescence reporter signal (DRn) versus cycle number during PCR.

**Figure 2.**
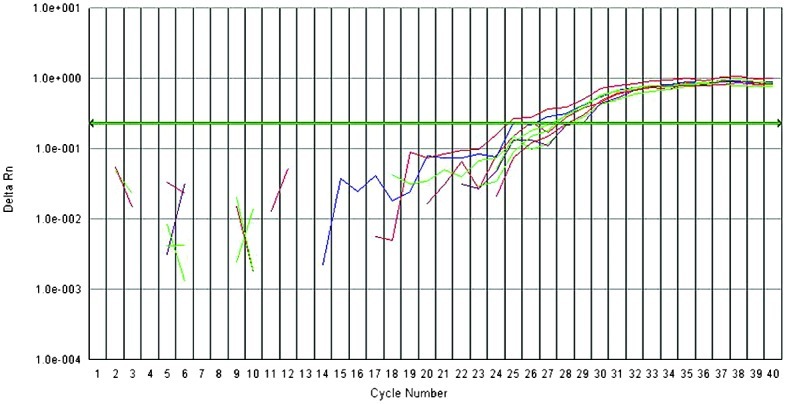
Fluorescent quantitative RT-PCR amplification curves of CD44v6. The graph of the increment of fluorescence reporter signal (DRn) versus cycle number during PCR.

**Figure 3.**
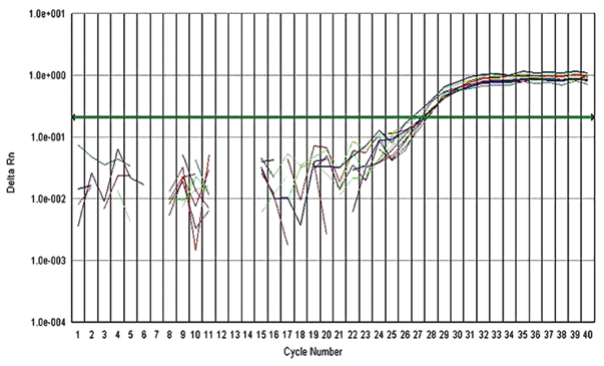
Fluorescent quantitative RT-PCR amplification curves of CK19. The graph of the increment of fluorescence reporter signal (DRn) versus cycle number during PCR.

Fluorescent quantitative PCR was used to detect the mRNA expression of CK19 and CD44v6 in the pulmonary venous blood before and after venous ligation. For each reaction tube, the fluorescence signal of the reporter dye (FAM) was divided by the fluorescence signal of the passive reference dye (ROX), to obtain a ratio defined as the normalized reporter signal (Rn). DRn represents the normalized reporter signal (Rn) minus the baseline signal. The results indicated that ΔCt values of CD44v6 and CK19 of NSCLC groups at the early period during surgery were 7.83±1.70 vs. 10.76±2.74, respectively, while those of the control group were 9.17±1.04 vs. 12.76±2.36. The mRNA expression of CD44v6 and CK19 in NSCLC groups was significantly higher than that of the control group (P<0.05) (Table I, Figs. 1–3).

### Patient characteristics and the expression of CD44v6 and CK19

There was a clear difference in CD44v6 mRNA level between squamous cell carcinoma and adenocarcinoma at late periods during surgery (7.95±1.81 vs. 5.81±2.23; P=0.008). No significant correlation was identified between the expression of CD44v6 and CK19 and any other clinicopathological characteristics, at early and late periods during surgery (Table II).

### Effects of different ligation sequences on the expression of CD44v6 and CK19

In the PA-first group, ΔCt values of CD44v6 and CK19 at early vs. late periods during surgery were 7.92±1.97 vs. 5.67±2.11 (P= 0.008) and 11.21±3.14 vs. 8.60±4.02 (P= 0.05), respectively. Both CD44v6 and CK19 values in the late period were significantly higher than those in the early period (P≤0.05). In the PV-first group, ΔCt values of CD44v6 and CK19 before and after ligation were 7.74±2.10 vs. 7.95±1.91, and 10.30±2.98 vs. 10.60±3.15, and ΔCt values of CD44v6 and CK19 before vs. after ligation were similar, exhibiting no statistically significant differences (P>0.05; Table III). The results indicated that mRNA expression of CD44v6 and CK19 in the PA-first group in the late period during surgery was significantly higher than that in the early period.

## Discussion

The basic principles for tumor surgery advise that all procedures should be performed gently and any touching or crushing of the tumor should be minimized to reduce (or even avoid) the possibility that tumor cells may escape from the solid tumor cell nests and enter the circulation, thus causing blood micrometastasis. According to Turnbull *et al* and Hayashi *et al*(5,6), the application of a non-contact technique in colon carcinoma operations could reduce the formation of CTCs and improve the survival rate of patients. It was highly recommended that surgeons treat the tumor gently and avoid turning over and squeezing the mass as much as possible.

There has been emerging evidence to suggest that CK19 mRNA detection is strongly associated with the presence of lung cancer metastases or recurrence, particularly after surgery (6). CD44v6, the v6 variant of CD44, is mainly expressed in a subset of adenocarcinomas and the expression of adhesion molecule CD44v6 could well reflect the invasiveness and adhesiveness of malignant tumor cells. In this study, NSCLC patients were randomly divided into PA-first and PV-first groups, and blood samples from the early and late periods during surgery were both drawn from the vein in order to make an objective comparison between the effect of either PA or PV first. According to traditional theories, it is clearly required that ligation of the PV should be performed prior to that of the PA during lobectomy, to prevent the occurrence of CTCs entering the systemic circulation via pulmonary venous blood. No significant differences were identified between the expression of CD44v6 and CK19 in PV-first group at early and late periods during surgery, while significant differences were revealed in the PA-first group with a higher expression of CD44v6 and CK19 in the early period. It suggested that the operative procedures may play a promoting role in CTCs shedding off from the primary tumor and ingressing into the pulmonary venous blood, and that ligation of the PV first during surgery may help prevent the dissemination of CTCs. Ge *et al* detected the pre-, intra- and post-operative mRNA expression of CK19/CEA in peripheral venous blood of 23 NSCLC patients using fluorescent quantitative PCR (7). All patients were divided into either PV-first or PA-first groups at random. Results revealed that the intraoperative mRNA expression of CK19 was significantly higher than the pre- or post-operative expression (P<0.05); the highest mRNA expression of CEA was identified after surgery (P<0.05) and the ratio of CK19/CEA in the PA-first group was higher than that in the PV-first group (but without statistical significance). Yamashita also revealed that video-assisted thoracoscopic surgery (VATS) lobectomy was associated with a higher risk of seeding of tumor cells into the circulation during operation (8). Based on these results, it appears that surgical manipulation is capable of promoting the release of tumor cells into the bloodstream, and ligation of the PV prior to ligation of the PA may partly prevent such release during surgery.

Although numerous studies appear to support the opinion that the PV should be ligated prior to the PA during surgical intervention and that this order of vessel ligation may improve the prognosis of patients with NSCLC, there are still various opinions. According to Refaely *et al*, different sequences of PA and vein ligations bore no correlation with tumor recurrence and prognosis (2). In the study, a total of 279 NSCLC patients were randomly divided into PV-first (133 patients, 48%) and PA-first (146 patients, 52%) groups. The follow-up results revealed that the total recurrence rates in the two groups were similar and multivariate analysis (controlling for the effect of the performing surgeon) revealed that sequence of vessel interruption was not a risk factor. The results revealed that the sequence of vessel interruption during lobectomy did not play a role in tumor recurrence.

It is conceivable that most of the cancer cells shed into the blood stream during surgery are ultimately likely be destroyed by natural defense mechanisms, and extremely few of the tumor cells succeed in establishing secondary tumors. As the content of tumor cells in the blood circulation was extremely low, the sensitivity and specificity of detection is commonly required so that even one tumor cell can be detected in 10^5^–10^7^ cells. At present, the detection methods for tumor micrometastasis include immunohistochemistry, flow cytometry and RT-PCR, among which RT-PCR has been well accepted by virtue of its high sensitivity and specificity in detecting one tumor cell among 10^5^–10^7^ cells (9,10).

Numerous studies confirmed that the detection of CTCs in peripheral blood was associated with poor prognosis of the patient. Using RT-PCR, Yamashita (11) analyzed CEA mRNA expression in peripheral blood from 103 patients with NSCLC at the time of diagnosis, prior to surgery, and 2–3 weeks later, following surgery. The results revealed that patients with high mRNA expression of CEA in the pre-operative blood samples had a poor survival rate when compared with those without CEA mRNA expression. Similarly, Yoon *et a*l (12) demonstrated that expression of CTCs in pulmonary peripheral blood was correlated with poor prognosis of the patient.

In this study, both CK19 and CD44v6 were used as markers to monitor the fluctuation in CTCs of patients. Whether micrometastasized cells in the blood of malignant tumor patients leads to the formation of metastatic foci depends on the invasiveness and adhesiveness of micrometastasized cells. CD44v6 is mainly expressed in a subset of adenocarcinomas and may be a useful marker for tumor screening. Studies demonstrated that the expression of adhesion molecule CD44v6 may reflect the invasiveness and adhesiveness of malignant tumor cells (13,14). Thus, the expression of CD44v6 in pulmonary venous blood was detected in this study, and the results revealed that the level of CD44v6 was closer to the experimental expectation than that of CK19. The expression of CD44v6 may be a good indicator of CTCs. However, this requires further investigation.

Our results suggested that ligation of the PV should be performed first during lobectomy. It was demonstrated that surgical manipulation itself may stimulate the occurrence of blood micrometastasis. Thus, ligation of the PV first during surgery may help to prevent blood micrometastasis. To clarify the clinical significance of different sequences of pulmonary vessel interruption, a prospective study with randomized selection of the method for each patient and with long-term follow-up should be conducted.

## Figures and Tables

**Table I.** Differences in mRNA expression between CD44v6 and CK19 in pulmonary venous blood of NSCLC groups and those in the control group.

Group	Number of cases	CD44V6	CK19
NSCLC	30	7.83±1.70	10.76±2.74
Control	10	9.17±1.04	12.76±2.36
P-value		0.006	0.039

NSCLC; non-small cell lung cancer.

**Table II.** Patient characteristics and the expression of CD44v6 and CK19.

		Early period				Late period			
Patient characteristics	No.	CD44v6	P-value	CK19	P-value	CD44v6	P-value	CK19	P-value
Gender			0.875		0.266		0.552		0.271
Male	14	7.78±2.13		11.43±3.04		7.08±2.46		10.41±3.52	
Female	16	7.89±1.93		10.17±3.02		6.57±2.16		8.90±3.81	
Smoking			0.890		0.293		0.241		0.133
Yes	20	7.86±2.28		9.83±3.56		6.41±1.97		8.88±3.66	
Non	10	7.77±1.40		11.22±2.73		7.61±2.77		11.04±3.49	
Histology			0.652		0.377		0.008		0.242
Squamous cell carcinoma	14	8.01±2.26		10.29±2.46		7.95±1.81		8.85±3.60	
Adenocarcinoma	16	7.67±1.82		11.29±3.62		5.81±2.23		10.46±3.75	
Site of primary tumor			0.965		0.142		0.082		0.133
Hilar	11	7.85±2.02		9.67±3.24		5.86±2.14		8.26±3.96	
Peripheral	19	7.82±2.05		11.38±2.82		7.36±2.24		10.38±3.40	
Pathological stage			0.331		0.496		0.059		0.159
I	9	8.47±2.06		11.51±3.25		7.96±1.43		10.59±3.67	
II	13	7.89±2.05		10.00±2.92		6.91±2.37		10.23±2.93	
IIIA	8	7.01±1.82		11.14±3.12		5.36±2.17		7.47±4.38	

**Table III.** Effects of different sequences of ligation during surgery on the mRNA expression of CD44v6 and CK19.

		CD44v6			CK19		
Group	Number of cases	Early period	Late period	P-value	Early period	Late period	P-value
PA-first	15	7.92±1.97	5.67±2.11	0.008	11.21±3.14	8.60±4.02	0.050
PV-first	15	7.95±1.91	7.74±2.10	0.558	10.60±3.15	10.30±2.98	0.532

PA, pulmonary artery; PV, pulmonary vein.
